# Trends in mortality from Hodgkin's disease in western and eastern Europe

**DOI:** 10.1038/sj.bjc.6600452

**Published:** 2002-08-01

**Authors:** F Levi, F Lucchini, E Negri, P Boyle, C La Vecchia

**Affiliations:** Cancer Epidemiology Unit and Cancer Registries of Vaud and Neuchâtel, Institut universitaire de médecine sociale et préventive, CHUV-Falaises 1, 1011 Lausanne, Switzerland; Laboratory of Epidemiology, Istituto di Ricerche Farmacologiche ‘Mario Negri’, Via Eritrea 62, 20157 Milano, Italy; Division of Epidemiology and Biostatistics, European Institute of Oncology, Via Ripamonti 435, 20141 Milano, Italy; Istituto di Statistica Medica e Biometria, Università degli Studi di Milano, Via Venezian 1, 20133 Milano, Italy

**Keywords:** Hodgkin's disease, mortality, trends, Europe

## Abstract

Hodgkin's disease mortality rates steadily declined by about 75% between the late 1960's and the late 1990's in the current European Union countries and the USA, and Japan. Eastern European countries, however, showed only an approximately 40% decline between the late 1960's and the early 1990's, and no further fall thereafter.

*British Journal of Cancer* (2002) **87**, 291–293. doi:10.1038/sj.bjc.6600452 www.bjcancer.com

© 2002 Cancer Research UK

## 

Effective integrated chemo- and radiotherapy has made Hodgkin's disease (HD) a largely curable disease (Hoppe *et al*, 1994; La Vecchia *et al*, 1991). Between 1960 and 1990 mortality from Hodgkin's disease declined by over 60% in western Europe, and even to a greater extent in the USA and Japan. However, in former non-market economy eastern European countries the fall has been only about 30% (Levi *et al*, 1995). Recent economical and social developments in several eastern European countries have been associated with a fall in total mortality (Zatonski and Boyle, 1996; Zatonski *et al*, 1998) as well as in mortality from curable neoplasm such as testicular cancer (Becker and Boyle, 1997; Levi *et al*, 2001).

## METHODS

We considered, therefore, recent trends in mortality from Hodgkin's disease in the European Union (EU, comprising Austria, Belgium, Denmark, Finland. France, Germany, Greece, Ireland, Italy, Luxembourg, Netherlands, Portugal, Spain, Sweden, UK), six eastern European countries providing data to the WHO mortality database (Bulgaria, the Czech Republic, Hungary, Poland, Romania, Slovakia), and, for comparative purposes, the USA and Japan. We obtained estimates of the resident population and numbers of deaths from the WHO Database, recoding the latter according to the Ninth Revision of the International Classification of Diseases. Age-adjusted rates, by 5-year age groups, were based on the world standard population (Doll and Smith, 1982). The methods have been discussed in detail elsewhere (Levi *et al*, 1999); however, mortality for HD in European countries has not been comprehensively examined.

## RESULTS

Figure 1

**Figure 1:**
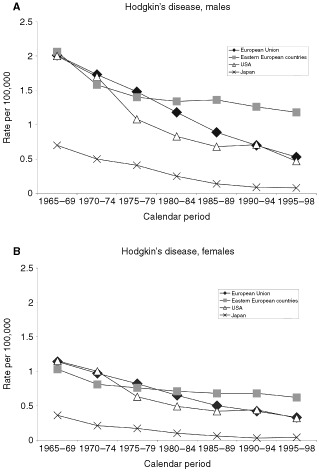
Trends in age-adjusted (world population) overall death certification rates from Hodgkin's disease per 100 000 men (**a**) and women (**b**) in the European Union, selected eastern European countries (Bulgaria, Czech Republic, Hungary, Poland, Romania and Slovakia), USA and Japan, from 1965–69 to 1995–98.

gives trends in mortality from HD in four broad geographic areas. In the late 1960's, mortality rates from Hodgkin's disease were very similar in the EU, eastern European countries and the USA, i.e. around 2/100 000 men and 1.2/100 000 women. Rates were appreciably lower (around 0.7/100 000 men, 0.4 for women) in Japan. In all the areas considered, substantial falls in mortality were observed, which were earlier and greater throughout the 1970's and 1980's in the USA as compared to the EU, and only in the late 1990's did HD mortality in the EU (0.53/100 000 men, 0.33 for women) approach that in the USA (0.47/100 000 men, 0.32 for women). The overall fall in men between 1965 and 1998 was 74% in the EU and 77% in the USA, and 71 and 72%, respectively, for women. Between the late 1980's and the late 1990's, the fall was 40% for males and 34% for females in the EU, 34% for males and 24% for females in the USA. Although Japan had very low rates in the 1960's, a further appreciable fall was observed, to reach rates of 0.08/100 000 men and 0.04 for women in 1995–98.

Eastern European countries showed an approximately 40% decline in HD mortality between the late 1960's and the early 1990's, to reach 1.26/100 000 men and 0.68 women. However, no appreciable further fall was observed over more recent years, and the rates were 1.18/100 000 men and 0.62 for women in 1995–98.

Table 1

**Table 1:**
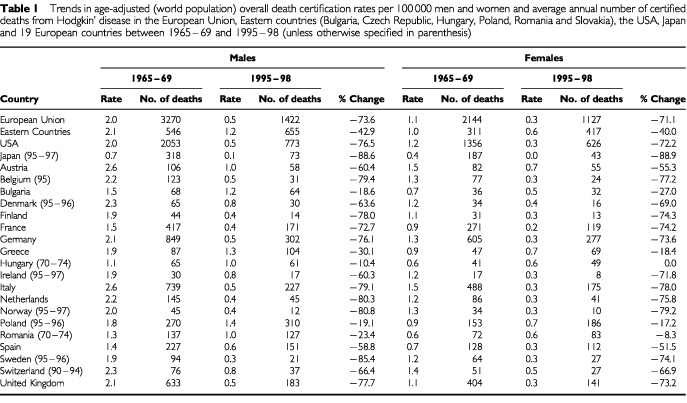
Trends in age-adjusted (world population) overall death certification rates per 100 000 men and women and average annual number of certified deaths from Hodgkin' disease in the European Union, Eastern countries (Bulgaria, Czech Republic, Hungary, Poland, Romania and Slovakia), the USA, Japan and 19 European countries between 1965–69 and 1995–98 (unless otherwise specified in parenthesis)

shows age-adjusted death rates from HD for men and women in 19 European countries besides the EU, USA and Japan in 1965–69 and 1995–98. In most western European countries, rates fell by 70–80% over the last three decades. However, the decline in HD mortality was much less (10 to 30%) in selected eastern European countries. About a third of patients dying from HD are aged 15 to 44 years. The pattern of trends in the young across most countries was similar to that for all ages. For men aged 15 to 44 the fall was 79% (from 1.9 to 0.4/100 000) in the EU, 77% (from 2.1 to 0.5/100 000) in the USA, and 82% (from 0.3 to 0.1/100 000) in Japan. Corresponding values for women were −79% (from 1.3 to 0.3/100 000) in the EU, −75% (from 1.2 to 0.3/100 000) in the USA, and −80% (from 0.2 to 0.04/100 000) in Japan.

## DISCUSSION

The present data indicate that HD mortality in the EU, the USA and Japan has continued to decline, though to a smaller degree, over more recent calendar periods, and hence confirms the persistence of advancements in the integrated treatment of HD. Rates are exceedingly low in Japan, whose HD mortality was already a third than those of North America and Europe in the 1960's, and is now less than a fifth than in other developed areas of the world. It is unlikely that differences in death certification validity and accuracy can explain such large differences.

The decline in HD mortality was similar in the older age groups, while for leukaemias the falls tend to be greater at younger age (Levi *et al*, 2000). This suggests that advancements in treatment for HD had a favourable impact also in the elderly.

In eastern Europe, there has been not only a substantial delay in the fall of HD mortality, but no progress has been observed during the 1990's, although efficacious chemotherapy schemes for HD (Hoppe *et al*, 1994), as well as radiotherapy, should, in principle, now be available in those countries, too. These unfavourable trends contrast with the declines in mortality from cardiovascular diseases and total mortality observed from the early 1990's onwards in Poland, Hungary, the Czech Republic and other major eastern European countries (Zatonski and Boyle, 1996 ; Zatonski *et al*, 1998). Thus, over a thousand potentially avoidable deaths per year from HD, largely at younger ages, with an associated loss of many decades of life expectancy, are still taking place in these countries every year.

As already observed for childhood leukaemia (La Vecchia *et al*, 1998; Levi *et al*, 2000) and testicular cancer (Levi *et al*, 2001), the fall in mortality for HD has been earlier and greater in North America than in western Europe. Even to a more dramatic extent than for childhood leukaemias or testicular cancer, the present data indicate that substantial inadequacies are persistent in the management of curable neoplasms in eastern European countries, pointing to the need for a rational organization of cancer control and treatment in those countries.
